# Sodium Valproate Enhances the Urethane-Induced Lung Adenomas and Suppresses Malignization of Adenomas in Ovariectomized Female Mice

**DOI:** 10.1155/2015/218219

**Published:** 2015-09-27

**Authors:** Donatas Stakisaitis, Raminta Mozuraite, Nomeda Juodziukyniene, Janina Didziapetriene, Saule Uleckiene, Paulius Matusevicius, Angelija Valanciute

**Affiliations:** ^1^Laboratory of Carcinogenesis and Tumor Pathophysiology, National Cancer Institute, Vilnius University, Santariskiu 1, LT-08660 Vilnius, Lithuania; ^2^Department of Histology and Embryology, Lithuanian University of Health Sciences, Mickeviciaus 9, LT-44307 Kaunas, Lithuania; ^3^Veterinary Academy, Lithuanian University of Health Sciences, Tilzes 18, LT-47181 Kaunas, Lithuania

## Abstract

In the present study, the possible effect of sodium valproate (NaVP) on urethane-induced lung tumors in female mice has been evaluated. BALB/c mice (*n* = 60; 4–6 weeks old, females) were used in the following groups: (1) urethane-treated; (2) urethane-NaVP-treated; (3) only NaVP-treated; (4) control. In the same groups, ovariectomized female mice (*n* = 60) were investigated. Urethane was given intraperitoneally, with a total dose of 50 mg/mouse. In NaVP-treated mice groups, 0.4% aqueous solution of NaVP was offered to mice* ad libitum*. The duration of the experiment was 6 months. The number of tumors per mouse in ovariectomized mice and in those treated with urethane and NaVP was significantly higher than in mice treated with urethane only (8.29 ± 0.58 versus 6.0 ± 0.63, *p* < 0.02). No significant difference in the number of tumors per mouse was revealed while comparing the nonovariectomized urethane- and urethane-NaVP-treated groups (*p* = 0.13). A significant decrease of adenocarcinoma number in ovariectomized mice treated with a urethane-NaVP as compared with ovariectomized mice treated with urethane only was found (*p* = 0.031). NaVP together with low estrogen may have a protective effect on the malignization of adenomas in ovariectomized mice.

## 1. Introduction

Urethane-induced lung tumors in mice models were well characterized and accepted for human lung adenocarcinoma investigations [1]. BALB/c mice are considered as susceptible to the development of lung tumor by urethane, and tumors can be modified by the influence of modulatory agents [2, 3]. Recently, it has been shown that NaVP, used together with urethane, synergistically enhances urethane tumorigenicity in lungs of only noncastrated male BALB/c but not in castrated male mice [4]. NaVP increases the turnover of gamma-aminobutyric acid (GABA) and thereby potentiates GABAergic functions [5]. The GABA-A receptor is rapidly activated by NaVP in cells [6]. GABA may affect cancer growth by activating GABA receptors. The gene expression investigation of GABA-A and GABA-B receptors in tissues of non-small cell lung cancers (NSCLC) showed that the gene expression of GABA receptor phenotypes was correlated with gender-related differences in cancerogenesis and clinical prognosis [7]. The GABA-A receptor is an ionotropic receptor; its subunits form a functional chloride channel [8, 9]. NaVP has been shown to enhance the gender-related urinary excretion of chloride in rats [10]. The GABA-A receptor subunits are expressed in rat kidney proximal convoluted and straight tubules and in other tissues [7, 11, 12]. Additionally, NaVP defines a class of histone deacetylases (HDAC) inhibitors that induce the modulation of tumor growth properties in clinical and preclinical studies [13–15].

The present study was designed to determine whether there are differences in lung tumorigenesis in a urethane-induced mouse lung tumor model in ovary-intact and ovariectomized mice and in the effect of NaVP on tumorigenesis in respective BALB/c female mice groups. The study data have shown that NaVP together with urethane increases tumorigenicity in ovariectomized mice but not in intact urethane-treated female mice, while a significant decrease of adenocarcinoma number in ovariectomized mice treated with urethane and NaVP as compared with ovariectomized treated with urethane only was found. NaVP may have a protective effect on the malignization of mice adenomas in ovariectomized rats.

## 2. Materials and Methods

### 2.1. Animals

Female BALB/c mice (*n* = 60; 5–7 weeks old) and ovariectomized BALB/c female mice of similar age (*n* = 60) were used in the study. The animals were purchased from the Animal Facility of Veterinary Academy, Lithuanian University of Health Sciences (Kaunas, Lithuania). The experiments were performed in compliance with the relevant laws. The permissions of the State Food and Veterinary Service of Lithuania to use experimental animals for research were obtained (no. 0177/30/04/2008/2 and 25/07/2013). Mice ovariectomy was performed by removing ovaries at the age of 5-6 weeks. The animals were housed in standard colony cages with free access to food and acclimatized for one week before the study; they were housed under conditions of constant temperature (21 ± 1°C), humidity, and the light/dark cycle (12 h/12 h). A commercial pellet diet was provided* ad libitum*.

### 2.2. Experiment Protocol

Intact-ovary female mice were randomly divided into four groups (*n* = 15 in a group): (1) treated with urethane, (2) treated with urethane and NaVP, (3) treated with NaVP only, (4) intact control. In the same four groups (*n* = 15 per group) ovariectomized female mice were investigated. In the urethane-treated groups, 10 mg urethane/mouse in 0.2 mL sterile physiological solution was given twice a week by intraperitoneal injections (5 doses, a total dose of 50 mg/mouse). The second and third groups of mice were treated* per os* with NaVP 0.4% aqueous solution (in the second group starting week prior to urethane administration) for six months. The NaVP solution which was their only source of drinking was offered to animal* ad libitum* (in aluminum foil-wrapped bottles to avoid light decomposition). Controls and urethane groups were given fresh drinking water provided* ad libitum*.

NaVP was from Sigma-Aldrich Chemie GmbH, Germany, and urethane (ethyl carbamate) was from Fluka.

### 2.3. Macroscopic Examination

After 6 months, animals were killed in a 70% CO_2_ camera. Lungs were collected with tracheas and together with hearts were fixed in a 10% neutral buffered formalin solution for 2 weeks and taken for macroscopic evaluation. The percentage of animals with lung tumors, the mean number of tumors per tumor-bearing mouse, and tumor growth (examination of tumors in subgroups according to the diameter of tumor in the groups: <1 mm, 1 to 2 mm, and >2 mm) were considered in each group of animals only in mice that survived up to the end of the experiment.

### 2.4. Histological Examination

After macroscopical examination, lungs were divided into lobules, paraffin blocks were made, and 3 *μ*m sections were performed with a LEICA RM 2155 microtome. Standard hematoxylin-eosin staining was used. Histological slides were evaluated using the OLYMPUS BX 40F4 microscope supplied with a digital XC30 camera. Magnification 4 × 10 was used to evaluate the presence of a tumor in the lobule. Magnification 40 × 10 was used to evaluate tumor type and malignancy. Histological investigation was performed by two independent researchers. Mice lung tumors according to their morphology were divided into two groups: (1) benign adenoma and (2) malignant adenocarcinoma. Such division of urethane-induced mice lung tumors is widely used in scientific investigations [16, 17]. We evaluated the shape of the nuclei, nuclear pleomorphism, cell size, different ratio between the cytoplasm and nucleus amount, an increased number of nucleoli, different nucleoli shape, and a changed intensity of cytoplasm staining [18].

### 2.5. Statistical Analysis

The results were counted and analyzed using the SPSS 20 (IBM) statistical program using means ± standard error of the mean (SEM). Student's *t*-test was used to find differences between groups. Differences at the value of *p* < 0.05 were considered significant. The Mann-Whitney test (median; range) was used to calculate the significance of differences in adenoma and adenocarcinoma scores between the studied groups. The Wilcoxon signed-rank test was used to compare the number of adenocarcinoma tumors with adenoma tumors.

## 3. Results

### 3.1. Mice Survival in the Study Groups

*Nonovariectomized Mice.* Mice in control and NaVP-treated groups survived until the end of the experiment (Table 1). In the female urethane-treated group, five mice did not survive: three mice died during the 1st–3rd months, one died during the 4th month, and one died during the 5th month of the experiment. In the urethane-NaVP-treated group, two mice died (one in the 1st month and the other in the 5th month). 

*Ovariectomized Mice.* All mice survived in the NaVP-treated group. Up to six months of experiment, 12 mice survived in the control (one mouse died in the 2nd month and two in the 4th month), 12 mice died in urethane-treated group (one mouse died in the 3rd month and two in the 4th-5th months), and 14 mice died in the urethane-NaVP-treated group (one mice died in the 2nd month).

Survival data imply that young animals are sensitive to urethane and urethane-NaVP toxicity. Autopsies of mice that perished during the 5th month revealed that, in urethane- and urethane-NaVP-treated groups, lung tumors had developed in all cases, but data on these animals were not taken for statistical assessment.

### 3.2. Differences in the Number of Lung Tumors per Mouse between Intact and Ovariectomized Mice Groups

**Figure 1:**
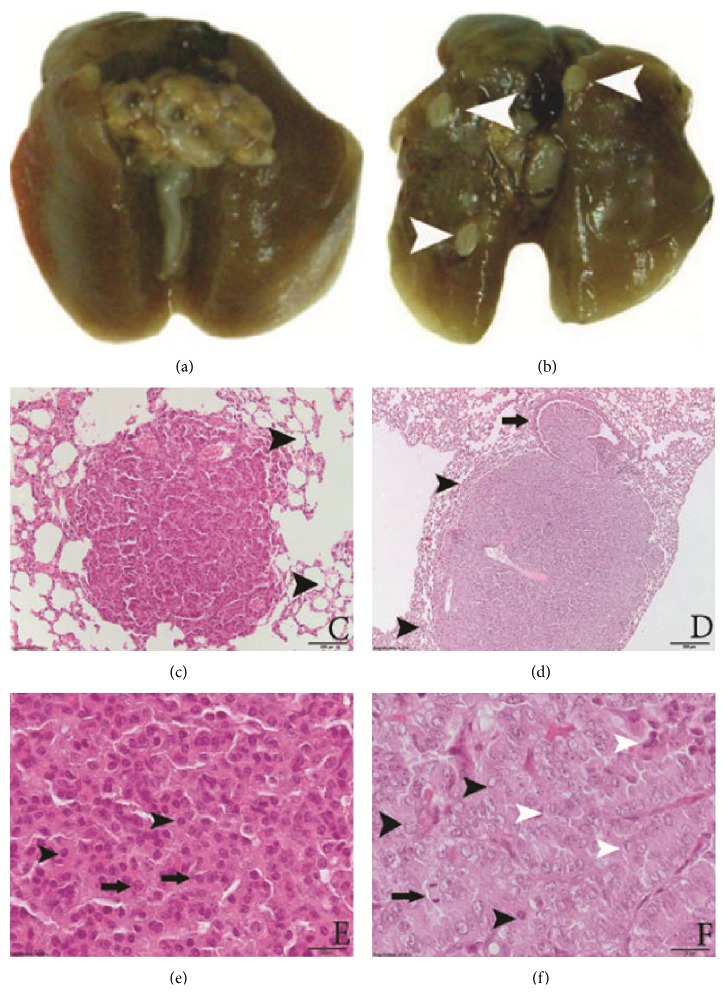
Lung specimens. (a) Control. (b) Urethane-induced lung tumors (white arrowheads). (c) Adenoma, magnification ×4, H & E staining. There are no signs of pressure to surrounding tissues; alveoli are well preserved (black arrowheads). (d) Adenocarcinoma, magnification ×4, H & E staining. Compression of surrounding alveoli (black arrowheads). Invasion to the lumen of bronchiole (black arrows). (e) adenoma, magnification ×40, H & E staining. All nuclei are of similar size and shape; no nucleoli are present (black arrowheads). Cells are of similar size and shape (black arrows). (f) Adenocarcinoma, magnification ×40, H & E staining. Pleomorphic nuclei (black arrowheads), mitotic figures (black arrow), and different cell shapes and sizes (white arrowheads). Scale bar: (c and d) 200 *μ*m; (e and f) 20 *μ*m.

Mice with lung tumors were found neither in intact control nor in the intact NaVP-treated and analogous ovariectomized mice groups. All urethane-treated mice of both groups (ovary-intact and ovariectomized) developed tumors (Table 1). Urethane-caused tumors may be found in any lobe of a lung and are often situated just beneath the pleura and are recognized by their nodular, pearly, and grey-white appearance contrasting with the more pink color of the normal lung parenchyma (Figures 1(a) and 1(b)).

**Figure 2:**
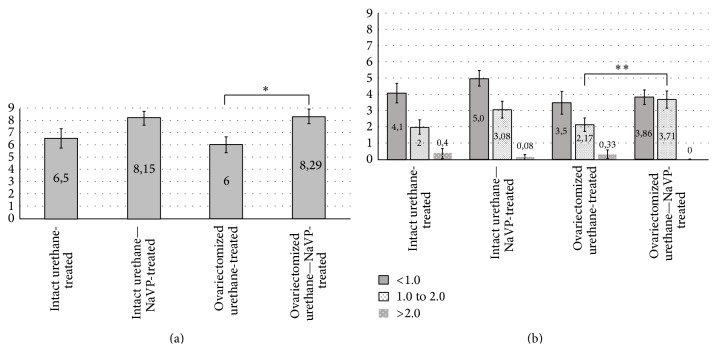
Number of tumors in intact and ovariectomized mice groups. (a) Total number of tumors on the surface of the lungs. ^*∗*^ *p* < 0.02 in comparison with the ovariectomized urethane-treated group. (b) Tumors in accordance with tumor diameter. ^*∗∗*^ *p* < 0.05 in comparison with the ovariectomized urethane-treated group.

When comparing the number of tumors per mouse in intact urethane-NaVP treated and urethane-NaVP-treated ovariectomized mice, lung tumors were found statistically significantly more often in urethane-NaVP-treated ovariectomized females (6.5 ± 0.79 versus 8.29 ± 0.05, *p* < 0.02; Table 2, Figure 2(a)).

No significant difference in the number of tumors per mouse was detected when comparing intact urethane-treated and ovariectomized urethane-treated female groups and in separate subgroups of these groups according to the diameter of tumors (Table 2; *p* > 0.2, Figure 2(b)).

No significant difference in the number of tumors per mouse was revealed also while comparing the intact urethane- and intact urethane-NaVP-treated groups and in separate subgroups of these groups according to the diameter of tumors (*p* > 0.05; Table 2, Figure 2(b)).

The data show that NaVP in ovariectomized mice treated with urethane caused an increase in the number of tumors per mouse in the subgroup with the tumor diameter 1.0 to 2.0 mm as compared with urethane-NaVP-treated intact-ovary mice (*p* < 0.05; Table 2, Figure 2(b)).

### 3.3. Frequency of Adenoma and Adenocarcinoma in Intact-Ovary and Ovariectomized Mice Groups

According to morphology (microscopical investigation of lung lobules), tumors were divided into adenomas and adenocarcinomas. In adenomas, we observed eosinophilic tumor cells, their structure was similar to that of hyperplasia, mitosis was very rare, and nuclear pleomorphism was absent. There was no invasion into surrounding tissues; alveoli were well preserved (Figures 1(c) and 1(e)). In adenocarcinomas, we observed the pressure of the surrounding tissues and infiltration into the bronchiolar lumen and wall (Figure 1(d)). The nuclei of tumor cells were pleomorphic; cells of different size and shape could be spindle-shaped atypical with frequent mitosis (Figure 1(f)). After evaluation of tumor type, adenocarcinomas and adenomas were counted, and data are presented in Table 3.

There was no significant difference in the number of adenomas and adenocarcinomas between treated with urethane and with urethane-NaVP nonovariectomized mice. Ovary-intact females exhibited a higher number of adenocarcinomas than ovariectomized mice did: we found a significant decrease of adenocarcinoma numbers in ovariectomized urethane-NaVP-treated mice as compared with ovariectomized animals treated with urethane only (*p* = 0.031; Table 3).

Comparing the median value of adenocarcinoma with the median value of adenoma, the number of adenocarcinomas was found significantly more often in the intact urethane-treated group (*p* = 0.024; Table 3) as well as in the intact urethane-NaVP-treated group (*p* = 0.041). Comparing the adenocarcinoma number (median value) with the adenoma median value in the ovariectomized urethane-treated group, adenocarcinomas were found significantly more often than adenomas (*p* = 0.006), and no significant difference was found in the ovariectomized urethane-NaVP-treated group (*p* = 0.671; Table 3).

## 4. Discussion

Female gender has long been observed to be a positive prognostic factor regardless of the lung cancer type, stage, and therapy. Whether these differences are of a biological, behavioral, or environmental nature remains unclear [19–21]. Sex differences in mouse models of lung cancers were reported [22]. The importance of lung cancerogenesis in both spontaneous and carcinogen-induced gender-related pulmonary cancers and spontaneous pulmonary adenomas in mice is determined by multiple genetic loci [23]. Urethane specifically initiates the development of lung tumors from airway epithelial cells in mice [1]. BALB/c mice are considered susceptible to the development of lung tumor by urethane [2].

The study data show that NaVP causes a significant increase of tumor number in ovariectomized urethane-NaVP-treated BALB/c female mice as compared to ovariectomized only urethane-treated mice, but a significant decrease of adenocarcinoma numbers in ovariectomized mice treated with urethane plus NaVP was found as compared with ovariectomized animals treated only with urethane.

Many aspects of the contribution of the NaVP pharmacological mechanisms and their significance in gender-related tumorigenesis have not been investigated. NaVP increases the GABA turnover and potentiates the GABAergic functions, reducing the activity of the GABA-degrading enzymes [5, 24]. The effect of NaVP on cellular mechanisms may be sex-specific with the differential response to basal GABA levels in males and females [25]. The GABA-A receptor is rapidly activated by NaVP in cells [6]. The GABAergic neuronal activity was about twofold higher in male than female rats [26]. A comparison between intact female rats and castrated animals indicated that endogenous GABA release in ovariectomized rats was only 60–70% of that in intact animals, while basal GABA levels are also significantly reduced in estrogen-treated ovariectomized rats [27]. In male rats, castration decreases the activity of GABAergic neurons, suggesting that GABAergic neurons are tonically stimulated by testosterone. Testosterone replacement prevents the castration-induced decrease in GABA [28].

The specificity of NaVP for GABA suggests that this interaction may be an important mechanism for NaVP pharmacological effects on cellular immunology. Lymphocytes have a functional GABAergic system which may operate as a modulator of T-cell activation [29]. GABA-A receptor expression in human peripheral blood mononuclear cells is regulated by gender [30]. Different subtypes of T cells, CD4 and CD8, from human, rat, and mice lymph nodes have been also shown to express mRNA and protein for subunits and had functional GABA-A channels [31]. NaVP has inhibited the proliferative capacity of T-lymphocytes by diminishing the* gl. thymus* weight and inducing a differentiation of thymic medullar epithelial cells into Hassall's corpuscles. The diminishing of the* gl. thymus* weight under the influence of NaVP was significant in castrated male rats. Gender differences in the Hassall corpuscles development in* gl. thymus* were noted in castrated rats [32]. The GABA interaction with the channels may affect immune response, but the underlying mechanisms and relevance of GABA signaling in the immune system of tumorigenesis is still not clear.

The GABA-A receptors are also found in nonneural cells, with cancerous cells included [7, 12]. The GABA-A receptor *θ* subunit (GABRQ) forms a functional ionotropic chloride channel [8, 9]. Such extrasynaptic receptors have a high affinity for GABA and open the chloride channels at low ambient GABA concentrations. This leads to changes in the membrane potential [33]. Opening the GABA-A receptor chloride channels lead to chloride efflux and cell membrane depolarization [34]. The plasma cell membrane potential influences cell proliferation [35]. GABA has been shown to regulate the proliferation of several cell types including stem cells [36], cortical progenitor cells [37], immune cells [38, 39], and mouse chondrogenic ATDC5 cells [40].

The high level expression of the GABA receptor gene in NSCLC tissues compared with the paired noncancerous ones implicated that the GABA, GABA-B, and GABA-A receptor pathways could be an important factor in NSCLC cell proliferation regulation. NSCLC patients with a high gene expression of the GABA-B receptor subunit 2 and a low expression of the GABA-A receptor subunit A3 had a significantly better prognosis, and the GABA treatment suppressed the proliferation of NSCLC cells* in vitro* via the GABA-B receptor [7, 41]. A correlation between the high expression of the GABA-B receptor subunit 2 gene and the greater survival rate in females with NSCLC was found [7].* In vitro* data show that the high-level GABA-B gene expression is associated with the inhibition of cancer cell proliferation [42–44]. Furthermore, the GABA-A receptor subunit A3 gene overexpression was found in NSCLC tissues [7, 45]. The high level GABA-A receptor subunit A3 gene overexpression is correlated with cancer cell development, and these patients had a worse outcome [12, 46, 47]. The higher gene expression of the GABA-A receptor subunit A3 was mostly detected in male patients who had a worse prognosis [7]. It is possible to suggest that tumor-stimulating effects of GABA-A receptor whether or not GABA itself or NaVP have tumor promotion or inhibiting effects will depend on the expression levels of GABA-A versus GABA-B receptors, which could be affected by sex hormones. The GABRQ is overexpressed in hepatocellular carcinoma cells but not in a normal cell line and GABA in the liver promotes the proliferation of cancer cells through GABRQ [12].

The study data indicate that NaVP in ovariectomized BALB/c mice decreases the progression of urethane-induced mice lung adenomas to adenocarcinomas. Lung tumor progression in mice was sensitive to estrogen, indicating that the tumor grade was higher in ovary-intact than ovariectomized mice [61]. Small airway epithelial cell-derived adenocarcinoma is the most common human lung cancer and is particularly prevalent in women. The proliferation of immortalized human small airway epithelial HPL1D cells is stimulated by a single dose of the tobacco carcinogen NNK via cAMP signaling downstream, and estrogen enhances this response. GABA blocks this cooperative signaling of NNK and of estrogen in HPL1D cells [48]. GABA inhibits tumor growth in mouse models of non-small cell lung cancer [41]. The effects of GABA and GABA receptors show that GABA-associated pathways could act positively or negatively in regulating cancer cell behavior. The injection of a combination of estrogen and progesterone produced a greater reduction in GABA-A receptor binding in mouse forebrain membranes, indicating that gonadal steroids contribute to the modulation of GABA-A receptor binding in male and female mice cells [49].

There could be other factors that may contribute to the NaVP gender-related induction of cell proliferation and tumorigenesis responses, for example, the link of GABA mechanisms with the ion cotransporter activity expression regulating the intracellular chloride concentration. The depolarizing effects of GABA are promoted by the relative accumulation of chloride inside the cells, leading to chloride efflux once GABA-A receptor channels open. Intracellular chloride regulation and the control of GABA-A receptor signaling are effected through K-Cl cotransporter [50]. To counteract their effects, the K-Cl cotransporter exports these ions thus decreasing intracellular chloride [51]. In male rat neurons, the K-Cl cotransporter is less expressed than in female [52]. The possible mechanisms of K-Cl cotransporter activity were demonstrated in the modulation of tumor development and progression [53]. NaVP has been shown to enhance the urinary excretion of chloride in rats of both genders, but the 24-hour chloriduretic response in male was significantly higher than in female rats [10]. Chloride is recognized to have an important role in tumorigenesis: the intracellular chloride concentration would be one of the critical messengers in cell growth/proliferation and differentiation processes [54–56].

NaVP is recognized as a novel class of HDAC inhibitors that induce the differentiation of transformed cells and show antitumor properties in clinical and preclinical studies by modulating multiple pathways including cell cycle arrest, cell differentiation, and apoptosis [13, 14, 57, 58]. Recent data indicate that HDAC play a dual role in tumorigenesis: oncosuppressive in the early stages and oncogenic in established tumor cells in mice models [15].

Increased tumor expression of the estrogen receptor *α* (ER*α*) was a negative prognostic factor in non-small cell lung cancer, while the absence of ER*β* tumor expression was also a negative prognostic factor [59]. Estrogen acts to promote the development of lung adenocarcinoma in mice, and this may be related to the activation of signaling pathways in which an ER plays an important role [60]. Estrogen functions via the receptor variants ER*α*/*β*. The ER*β* is the main ER in the lungs of mice, and estrogen promotes tumor progression in the mouse model of lung adenocarcinoma [61, 60]. In the Er*β* knockout (−/−) mouse, female but not male offspring were protected against development of lung tumors after in utero exposure to the polycyclic hydrocarbon dibenzochrysene [62].

The serum concentration of estradiol of female mice was significantly decreased by ovariectomy, while the concentration of testosterone was slightly increased, as secretion from adrenal cortex may be augmented in ovariectomized female mice [63, 64]. It was shown that the number of lung tumors per mouse was significantly more frequent in the noncastrated male mice group treated with urethane in combination with NaVP as compared with the only urethane-treated male group. This allowed to hypothesize that NaVP in the noncastrated urethane-treated BALB/c mice could act synergistically with urethane and testosterone, and testosterone is as an oncogenic factor in males in this model. Such effect of NaVP was not found in the urethane-treated castrated BALB/c male mice [4]. The present study pointed to the possibility that urethane-induced lung carcinogenesis in females may be inhibited by interaction of low level of estrogen and NaVP effect. Estrogen plays an important role in lung development, particularly in females [65], and is likely responsible for the greater susceptibility of women to chronic pulmonary disease, lung cancer, and the deleterious effects of carcinogens [66, 67]. ER*β* is the predominant form expressed in mouse lung [65]. Human lung tumors detected ER*β* in more than half of the tumors evaluated, whereas ER*α* was not expressed [68, 69]. Furthermore, studies elucidate a critical role for macrophages in promotion of urethane-induced lung carcinogenesis in mice [70]. The expression of *β*-estradiol was mainly localized in inflammatory cells in the lungs. Urethane elicits strong inflammatory reactions in response to cellular and DNA damage; macrophages have been described as being capable of expressing both ER*α* and *β*, with local estrogen production being part of the inflammatory reaction in lungs exposed to tobacco carcinogens [71].

It is known that the effect of urethane can be related to benign adenoma or adenocarcinoma lung tumorigenesis [1, 16]. The appearance of adenocarcinoma in the study model depends on the duration of exposure to urethane: such a model during up to 4 months is related to the development of lung adenomas which in the course of time develop into adenocarcinomas [1, 4, 16]. A deprivation of estrogens and treatment with NaVP slowed down the transformation of adenoma into adenocarcinoma in ovariectomized urethane-NaVP-treated mice.

## 5. Conclusion

In females, urethane-induced lung tumorigenesis was increased by NaVP treatment and in ovariectomized groups this increase was significant. The malignant transformation of lung proliferative lesions tended to be inhibited by a low estrogen and NaVP effect. The data allow to hypothesize that NaVP together with sex hormones, by changing the GABA-A receptor activity and the intracellular chloride level and by modulating HDAC, plays an important gender-related role in urethane-induced lung tumorigenesis in the BALB/c mice model. Additional experiments are ongoing to investigate gender-related differences of the urethane-NaVP effect in lung adenocarcinoma pathogenesis in the BALB/c mice model.

## Figures and Tables

**Table 1:** Incidence of lung tumors in female BALB/c mice of the study groups.

Groups of mice	Initial mice number (*n*)	Mice data after 6 months		
		*n*	Body weight m ± SD (g)	Mice with tumors (%)
Intact control	15	15	24.3 ± 1.1	0
Ovariectomized control	15	12	24.0 ± 3.6	0

Intact U-treated	15	10	21.4 ± 2.9	100
Ovariectomized U-treated	15	12	22.8 ± 3.9	100

Intact U-NaVP-treated	15	13	20.4 ± 3.7	100
Ovariectomized U-NaVP-treated	15	14	21.9 ± 3.7	100

Intact NaVP-treated	15	15	20.0 ± 3.2	0
Ovariectomized NaVP-treated	15	15	25.4 ± 2.4	0

**Table 2:** Incidence of lung tumors in castrated BALB/c female mice of the study groups in accordance with the tumor diameter.

Groups	Data of mice after 6 months				
	*n*	Tumors per mouse in accordance with tumor diameter (m ± SEM)			
		All	<1.0 (mm)	1.0 to 2.0 (mm)	>2.0 (mm)
Intact control	15	0	0	0	0
Ovariectomized control	12	0	0	0	0

Intact urethane-treated	10	6.5 ± 0.79	4.1 ± 0.6	2.0 ± 0.45	0.4 ± 0.31
Ovariectomized urethane-treated	12	6.0 ± 0.63	3.5 ± 0.69	2.17 ± 0.41	0.33 ± 0.26

Intact urethane-NaVP-treated	13	8.15 ± 0.55	5.0 ± 0.47	3.08 ± 0.51	0.08 ± 0.08
Ovariectomized urethane-NaVP-treated	14	8.29 ± 0.58^*∗*^	3.86 ± 0.44	3.71 ± 0.53^*∗∗*^	0.71 ± 0.29

Intact NaVP-treated	15	0	0	0	0
Ovariectomized NaVP-treated	15	0	0	0	0

^*∗*^ *p* < 0.02 in comparison with the ovariectomized urethane-treated group.

^*∗∗*^ *p* < 0.05 in comparison with the ovariectomized urethane-treated group.

**Table 3:** Number of adenomas and adenocarcinomas in experimental mice groups.

Group	*n*	Number of tumors					
		Adenocarcinoma		Adenoma		Adenocarcinoma + adenoma	
		Median (range)	Total	Median (range)	Total	Median (range)	Total
Intact control	15	0	0	0	0	0	0
Ovariectomized control	12	0	0	0	0	0	0

Intact urethane-treated	10	2 (0–6)^*∗∗*^	25	0 (0–2)	5	4 (1–7)	30
Ovariectomized urethane-treated	12	4 (1–6)^‡^	44	1 (0–3)	15	5.5 (2–7)	59

Intact urethane-NaVP-treated	13	4 (0–10)^*∗∗∗*^	47	1 (0–4)	18	6 (1–11)	65
Ovariectomized urethane-NaVP-treated	14	2.5 (1–5)^*∗*^	33	1 (0–7)	28	4 (1–10)	61

Intact NaVP-treated	15	0 (0-1)	1	0	0	1 (0-1)	1
Ovariectomized NaVP-treated	15	0	0	0	0	0	0

^*∗*^ *p* = 0.031 in comparison with ovariectomized urethane-treated (Mann-Whitney test).

^*∗∗*^ *p* = 0.024 comparing the median value of adenocarcinoma number with the adenoma in the intact urethane-treated group.

^*∗∗∗*^ *p* = 0.041 comparing the median value of adenocarcinoma with the adenoma in the intact urethane-NaVP-treated group.

^‡^ *p* = 0.006 comparing the median value adenocarcinoma number with the adenoma in the ovariectomized urethane-treated group.
